# Supplementary material for: Ion Drift and Polarization in Thin SiO2 and HfO2 Layers Inserted in Silicon on Sapphire
Source: Nanomaterials (Basel). 2022 Sep 28;12(19):3394. doi: 10.3390/nano12193394 (PMC9565775; doi:10.3390/nano12193394)
Supplement: Supplementary file 1 [file nanomaterials-12-03394-s001.zip › nanomaterials-1887242-supplementary.pdf]

Supplementary Materials

Ion drift and polarization in thin SiO_2 and HfO_2 layers inserted in silicon on sapphire

Vladimir Popov, Valentin Antonov , Kirill Tolmachev , Andrey Miakonkikh , Elizaveta Smirnova , Konstantin Rudenko.*

Thin hafnium oxide (HfO_2) thin films were grown as an additional for SOS structures by the plasma-enhanced atomic layer deposition (PEALD) technique on a silicon substrate before the wafer bonding and Si layer transfer on it (**Figure S1**), according to the invention patent [S1]. The pare pair of the wet cleaned wafers were placed in the vacuum chamber and heated in the vacuum up to 200°C for SFS or 400°C for SOF structures in order to remove the physically absorbed molecules from the wafer surfaces before bonding and to suppress the large stress generation during the followed thermal splitting of hydrogen implanted Si wafers. Using implanted hydrogen transfer of only Si layers allows avoiding the defect generation in the high-k stack at the H implantation and subsequent thermal treatments.

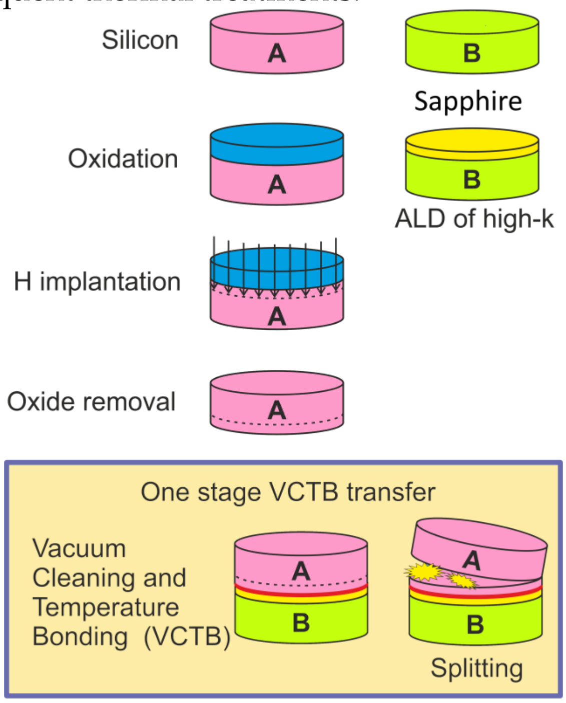

Figure S1. SOS structure fabrication process for the wafer #3 used in the investigation.

When the silicon and sapphire wafers were bonded together without the inserted layer, the 0.5-2.0 nm SiO_x interlayer growth was observed at the bonded interface after the high temperature (1000-1100°C) annealing in the inert atmosphere [5, Main text]. In the case of SiO_2 buried layer, the thermal silicon dioxide on the donor wafer A was not removed after hydrogen implantation for wafers #1 and #2 before the bonding with pure sapphire. Additionally, in order to decrease a fixed positive charge the nitrogen implantation with the fluences 3-5x10^{15} cm^{-2} in sapphire was used before boding for the wafer #1.

This wafer demonstrates typical transfer characteristics for SOS pseudo-MOSFETs, but asymmetrical shift relative $V_g = 0$ V due to the fixed negative charge near the Si/SiO_2 interface (Figure S2a). The mutual sweep with different sweep rates does not demonstrate the I_{ds} current hysteresis, but some slow relaxation was observed due to the charge trapping/detrapping at the interface states

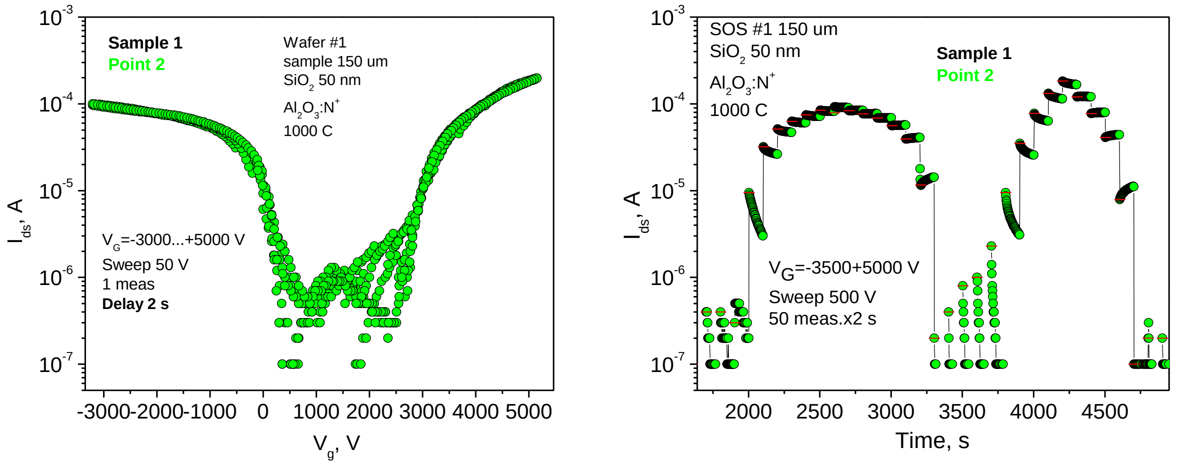

(**a**) (**b**)

Figure S2. (**a**) Transfer characteristics (drain current – gate voltage) for mutual V_g sweep between V_g = -3500+5000 V of SOS pseudo-MOSFET with 50 nm SiO_2 BOX layer and nitrogen implanted sapphire substrate; (**b**) the drain current I_{ds} evolution for sweeping bias voltage V_g during one unit sweep.

References

[26].Popov, V.P.; Zhanaev, E.D.; Dudchenko, N.V.; Antonov, V.A.; Popov, A.I. Method for Silicon-on Sapphire Formation. RF Patent No. 2538352; Byul. Izobret. No. 1, 2015. (In Russian).
